# Moving beyond the average: a method to measure health related-inequalities within randomised trials

**DOI:** 10.1186/s12939-026-02977-x

**Published:** 2026-09-29

**Authors:** Zachary D. V. Abel, Sophie Cole, Guido Erreygers, Laurence S. J. Roope, Philip M. Clarke

**Affiliations:** 1 Health Economics Research Centre, Nuffield Department of Population Health, University of Oxford, Oxford, OX3 7LF UK; 2 Department of Health Care Management, Technische Universität Berlin, 10623 Berlin, Germany; 3 National Institute for Health Research Oxford Biomedical Research Centre, John Radcliffe Hospital, Oxford, OX3 9DU UK; 4 Faculty of Business and Economics, University of Antwerp, Antwerp, 2000 Belgium; 5 Centre For Health Policy, University of Melbourne, Parkville, VIC 3010 Australia

**Keywords:** Health policy, Health inequality, Inequality in RCTs, Distributional effects

## Abstract

**Background:**

Randomised trials seldom investigate the impacts interventions have on socioeconomic inequality in health, focusing instead on their average treatment effects.

**Methods:**

We illustrate how standard measures of inequality such as the corrected concentration index can be reported in randomised experiments to quantify the interventions’ socio-economic distribution effect alongside more traditional average treatment effects. We illustrate this with a proof-of-concept example using a study on the impact of information and financial incentives on verified COVID-19 vaccination uptake in rural Ghana (*n* = 2,271). We estimate the distributional treatment effects of informational public health messaging, low- and high-cash financial incentives relative to a placebo group and present this information on an achievement plane.

**Results:**

Health messaging had a pro-rich socioeconomic inequality impact of -0.132 [95% CI: -0.28–0.02] relative to the placebo group, while the low and high cash incentives had impacts of -0.108 [95% CI: -0.29–0.07] and − 0.081 [95% CI: -0.26–0.11] respectively, providing no significant evidence for changes in inequality relative to the placebo group. A low-cash incentive was the only intervention that was shown to increase vaccination uptake.

**Conclusions:**

A socioeconomic inequality of health distributional indicator could routinely be calculated in experimental results and presented alongside average treatment effects. This would enable the identification of whether there are equality-efficiency trade-offs. The methods presented in this paper can be used to support policy decision making with representative datasets provided there is appropriate statistical power.

**Trial registration:**

The RCT investigated in this paper was registered with the American Economic Association on January 10, 2022, RCT ID: AEARCTR-0008775.

**Supplementary Information:**

The online version contains supplementary material available at https://doi.org/10.1186/s12939-026-02977-x.

## Introduction

Randomised experiments typically measure the average treatment effect of an intervention on a variable of interest. However, the socioeconomic distributional implications of interventions are rarely quantified in health system evaluation trials, which is surprising given that equity and equality are widely held principles in health systems [1]. Despite increased momentum to include distributional elements in economic evaluation, the practice is still not widespread [2–4].

One potential application is the evaluation of vaccination programs for diseases such as COVID, where socioeconomic health inequality indicators can complement conventional measures of effectiveness by quantifying how gains in coverage are distributed across socioeconomic groups. While within-country socioeconomic inequality in COVID-19 vaccine uptake during the pandemic has been documented, evidence remains limited, particularly in Low and Middle Income Countries (LMICs), with most studies lacking individual-level measures of socioeconomic status such as income or wealth, instead focusing on geographic level analysis [5]. Analysing 13 predominantly high-income countries using the Erreygers adjusted concentration index, Abel et al. (2025) found vaccine acceptance and uptake to be pro-rich in most settings, with the richest-poorest quintile gap reaching 30% points in France [6]. In Kenya, Niño et al. [7] similarly found a consistent pro-rich distribution of COVID-19 vaccination using the Erreygers index, with uptake ranging from under 6% in Garissa to over 46% in Nyeri [7]. These findings raise the question of whether interventions designed to increase uptake in low-income settings also reduce or exacerbate underlying health system inequities.

Duch et al. (2023) implemented a four-arm cluster-randomised controlled trial across 6,963 residents in six rural Ghanaian districts to assess how incentives and health messaging impacted on COVID vaccine uptake during the pandemic [8]. Villages were randomised to a placebo message, a standard health message, or low (20 cedis/~$3 US) or high (60 cedis/~$10 US) cash incentives. Cash incentive arms achieved vaccination intention rates of 81% versus 71% in the placebo arm, with the low cash incentive outperforming the high cash incentive.

Incorporating a socioeconomic health inequality indicator into trial reporting enables distributional considerations to be assessed alongside primary outcomes. In this paper, we propose a systematic approach for incorporating socioeconomic health inequality measures alongside average treatment effects in RCT reporting. While rank-dependent inequality indices such as the concentration index are well-established in health economics, their use as outcomes in randomised trials remains rare. We also explore how an achievement plane can be used to simultaneously visualise both average treatment effects and distributional treatment effects from an RCT. Using publicly available individual-level data from the Ghana Vaccine incentives trial we explore how inequality statistics can be calculated alongside measures of vaccine uptake, and use this as an application of proof-of-concept. We analyse the distributional treatment effects of public health messaging, low cash incentives, and high cash incentives on socioeconomic inequality in vaccine uptake, relative to a placebo group. These concepts can be applied with the necessary adjustments to any field concerned with the socioeconomic distributional impacts of interventions. Similarly, provided there is adequate power, the analysis can be extended to subgroups.

## Background

“To ensure healthy lives and promote well-being for all” is the third sustainable development goal, and many countries strive to provide equitable access to healthcare [1, 9]. If health systems strive to achieve equality in health outcomes, at a minimum, an understanding of how policies affect socioeconomic inequalities in health, which form an element of overall health inequality, is required. Whether income or wealth privileges individuals is an important aspect of such inequalities. Policy makers may be faced with trade-offs between maximising average health outcomes and reducing (or maintaining) socioeconomic inequalities in health outcomes, known as equality-efficiency trade-offs.

“Policy makers and researchers may be interested in features of the two marginal distributions and not simply the ATE (average treatment effect) . For example, if Y is disease burden, measured perhaps in QALYs, public health officials may be interested in whether a treatment reduced inequality in disease burden” Deaton & Cartwright, 2016 [10].

While randomised experiments are now often used to evaluate both health care and policy interventions, the focus is primarily on reporting the overall treatment effects. A study which randomly sampled 200 equity-relevant trials found that only 4% reported socioeconomic subgroup analyses whilst a further systematic review of infectious disease trials found that only 9% of trials reported socioeconomic status [11, 12]. In 2017, the CONSORT-Equity 2017 set out clear guidelines of how equity measures should be incorporated into equity-relevant trials [13].

Recently there has been increasing discussion of how to formalize taking inequality considerations into account through distributional cost-effectiveness analysis (DCEA) [14]. DCEA provides a framework to evaluate the health benefit/burden across socioeconomic groups in the general population. However, this method requires information on the socioeconomic distribution of health and this in turn requires a much greater understanding of the distributional impacts of interventions, which should ideally be based on evidence from randomised experiments.

The concentration index is one of the most widely used approaches to measuring socioeconomic inequality in health, summarising the degree to which a health variable is unequally distributed across the income distribution. Despite its widespread use as a descriptive measure, there has been little formal attention to causal inference using the concentration index. Ghasempour, de Luna and Gustafsson (2025) address this gap by defining a counterfactual concentration index at different levels of an exposure and deriving the conditions required for its identification [15].

A complementary route to causal inference is the direct integration of the concentration index as an outcome measure in randomised controlled trials. Because randomisation ensures that the socioeconomic distribution of participants is balanced across arms at baseline, any difference in the concentration index between the intervention and control groups at follow-up can be interpreted as a causal effect of the intervention on income-related inequality in the outcome. However, this approach has rarely been used: to the best of our knowledge, only one trial evaluating an intervention for low birthweight babies in India has reported concentration indices as an equity outcome measure [16].

In practice, researchers and policymakers are rarely interested in the overall effect of an intervention or its distributional impact in isolation. Finding ways to jointly characterise both average treatment and distributional impacts is therefore essential for a full evaluation of an intervention’s impact. The achievement index and achievement plane provide a natural framework for doing this, placing the average treatment effect and the change in the concentration index on a common footing and allowing the equity-efficiency trade-off to be visualised and assessed simultaneously.

The achievement plane and associated equity measures can, in principle, be built prospectively into trial design, e.g. through sample size calculations that explicitly account for the statistical power needed to detect distributional effects. However, until pre-specification of distributional outcomes becomes the norm, in practice the vast majority of analyses will need to be undertaken post-hoc, drawing on socioeconomic data that were collected for other purposes. Post-hoc application without adequate statistical power risks Type II errors, whereby a true distributional effect fails to reach significance. Researchers should therefore treat post-hoc analyses as exploratory rather than confirmatory, and exercise caution in interpreting non-significant differences in the concentration index as evidence of no equity effect. As the routine collection of socioeconomic status in trials becomes more common, there is a clear case for pre-specifying equity outcomes and powering studies accordingly, so that distributional questions can be addressed with the same rigour as average treatment effects.

## Methods

### A framework for considering distributional and average treatment effects

**Fig. 1:**
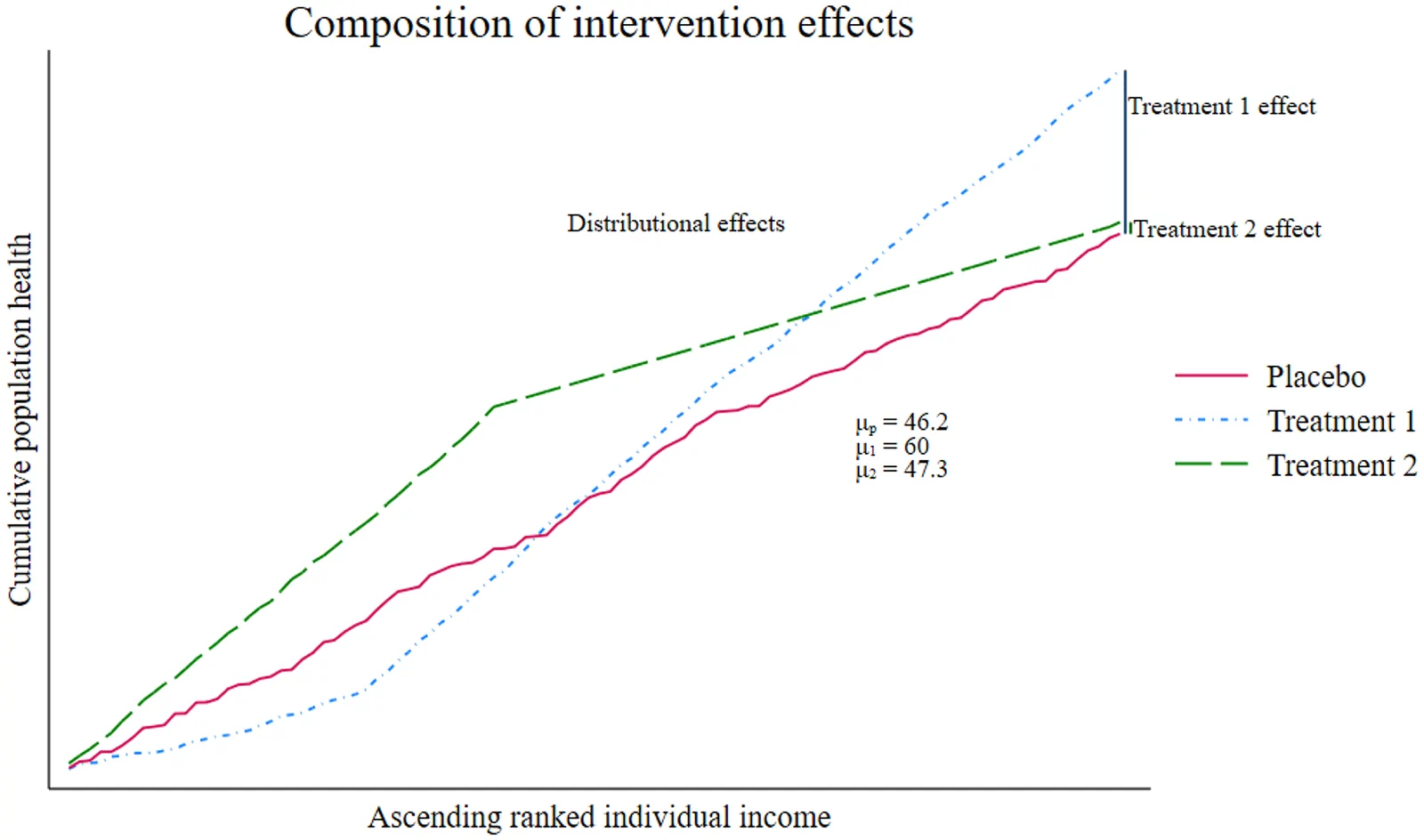
Composition of intervention effects in a hypothetical trial scenario

Consider a hypothetical intervention trial with three treatment arms as shown below in Fig. 1. We graph cumulative health of the populations against ascending ranked income, producing a Generalised concentration curve. Two effects are evident in the figure. The average treatment effect (ATE) is taken as the mean difference between the treatment groups (1 and 2) and the placebo group (p). ATE is given by (µ_*1*_- µ_*p*_) and (µ_*2*_- µ_*p*_) for treatments 1 and 2 respectively, where µ_*i*_ is mean uptake level of group *i*, and is the conventional effect noted in trials literature. However, there are also distributional treatment effects (DTE), reflected by the deviations of the treatment curves from the placebo curve. Despite a lack of meaningful difference in mean health between treatment 2 and the placebo, there is clearly a distributional effect benefitting the lowest-income earners. Treatment 1, however, has improved average health meaningfully, despite making the poorest in society worse off, constituting an equality efficiency trade-off. Both treatments lead to different socioeconomic inequality of health outcomes compared to the placebo, which warrants further measurement and investigation.

Socioeconomic inequality measures embody implicit value judgements. The choice of which measure to employ is partly subjective. Distributional comparisons depend on these choices, and particularly on the choice between absolute versus relative measures, which are conceptually very different and may give divergent results. Absolute measures consider absolute magnitudes of change, whereas relative measures consider proportional changes, relative to the starting position. Different absolute (and relative) measures can also lead to different conclusions, as they embody their own value judgements on what constitutes a desired distribution, or how sensitive we should be to inequality [17–19].

A simple summary measure of socioeconomic related inequality (e.g., the concentration index, generalised concentration index, and the extensions of these indices) can be estimated on the basis of the distributions presented above in Fig. 1.

### Data requirements

Incorporating a socioeconomic inequality of health measure requires data on the health outcome and a measure of socioeconomic status. The concentration index and its extensions are the most commonly used summary measures in health economics [20]. Respondent-level measures of socioeconomic status are preferred, such as individual income, education, or consumption, which are often collected as social determinants of health or as controls in trials. Where individual-level data are unavailable, area-level proxies such as neighbourhood deprivation indices or luminosity data can be used, provided sufficient within-sample variation exists; such measures are particularly useful in post-hoc applications [21, 22].

### Measuring socioeconomic health inequality

The concentration index, the workhorse of socioeconomic inequality analysis in health economics was first developed by Kakwani [23] and later introduced by Wagstaff et al. [24] It can be specified as follows:

1 $$\:C\left(h\right)\equiv\:1-\:\frac{{\sum}_{i=1}^{n}\left(2{\lambda\:}_{i}-1\right){h}_{i}}{{n}^{2}{\mu\:}_{h}}$$

where $\:{C}_{h}$ is the concentration index for health variable * h*; * n* is the sample size; $\:{\lambda\:}_{i}$ is the fractional rank of individual * i* in the socioeconomic distribution (i.e. the cumulative proportion of the population up to and including individual * i* when ranked by socioeconomic status from poorest to richest); $\:{h}_{i}\:$is the health variable for individual * i*; and $\:{\mu\:}_{h}$ is the mean of the health variable across the sample. The index takes a value of zero when health is equally distributed across the socioeconomic distribution, a positive value when health is concentrated among the better-off (pro-rich inequality), and a negative value when health is concentrated among the worse-off (pro-poor inequality). Several further extensions and adjustments have been produced to provide, for example, absolute measures of socioeconomic health inequality (Generalised concentration index, multiplied through by mean health levels [17]), measures fit for binary variables (Wagstaff index [25], and the Erreygers adjusted index, [26]), or to retain additional socioeconomic information (level index, [27]). Each is appropriate for different data or use cases, though relies on similar principles [27, 28].

The family of bivariate inequality measures can be used to discern summary differences in the distributions contained in Fig. 1 above.

### The equality efficiency trade-off

In the hypothetical example provided in Fig. 1, a policymaker may easily be able to select between the placebo and treatment 2, which offers similar mean health but improved health for the lowest-income earners. In this instance, treatment 2 dominates the placebo group in both equality (consistently above the placebo treatment) and efficiency (total above the placebo treatment). However, incorporating treatment 1 into the decision-making process illustrates that there may be trade-offs between the efficiency and equity impacts of an intervention as average (societal) health is improved at the expense of the lowest income earners.

In response to this trade-off, Wagstaff developed the achievement index: a measure incorporating both inequality between high and low-income earners, and average health levels [29]:

2 $$\:I=\:\mu\:\left(1-C\left(h\right)\right)$$

Multiplying out, this expression is $\:\mu\:-\mu\:C\left(h\right)$, which in turn is equivalent to $\:\mu\:-GCI$, where GCI is the generalised concentration index; the absolute version of the rank dependent concentration index [17], and the equivalent index of the Generalised concentration curves that are shown in Fig. 1. This work was extended by Clarke and Hayes who visualised the achievement index trade-off on a plane using change in (ill) health on the x-axis and change in absolute inequality on the y-axis to form an achievement plane [30].

**Fig. 2:**
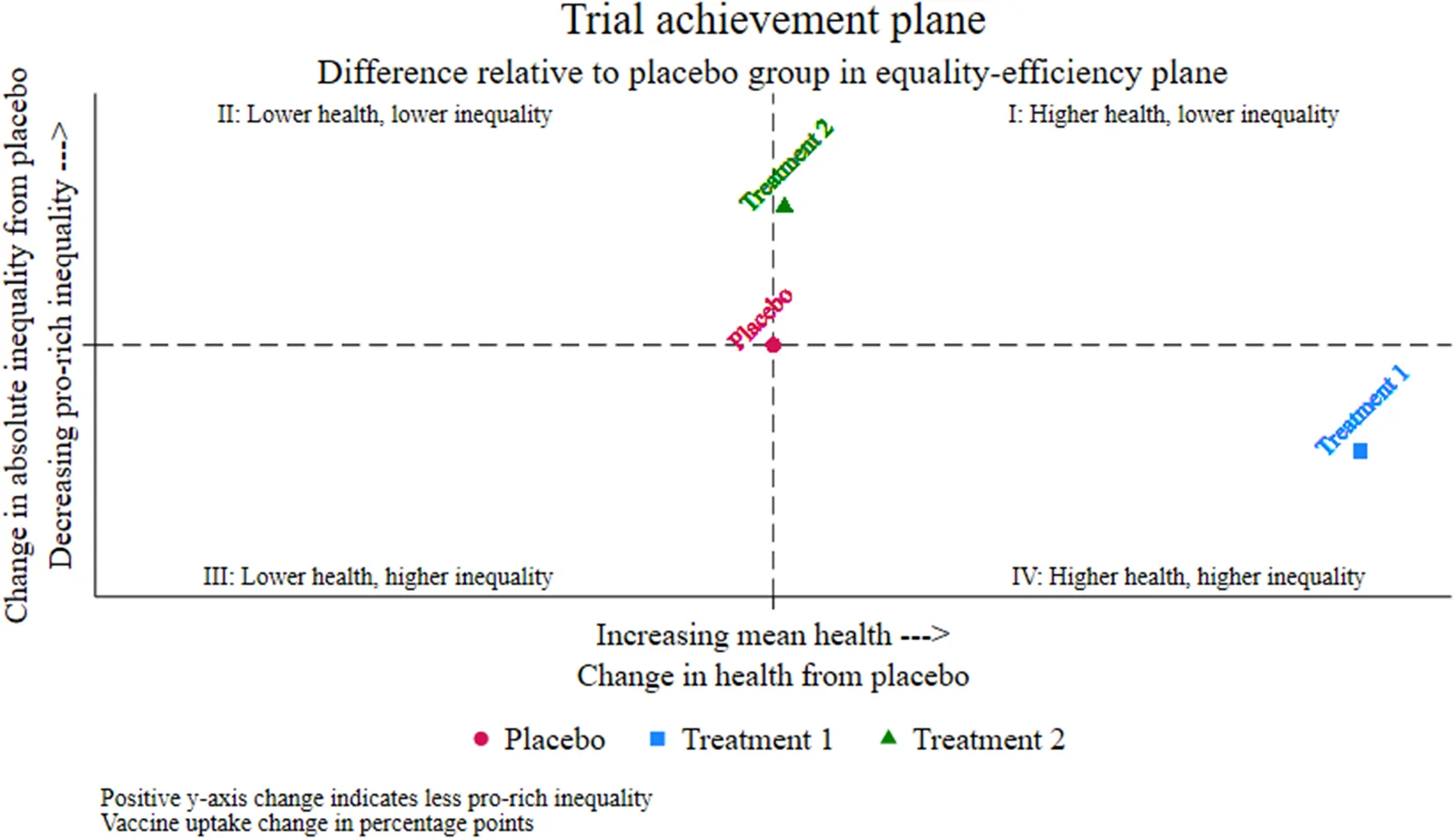
Hypothetical example of an evaluation trial achievement plane

We apply the principle of the achievement plane to evaluation trials, plotting the differences between treatment and placebo groups in an equality-efficiency space rather than against time. We show the mean difference in health on the x-axis $\:{(\mu\:}_{hi}-{\mu\:}_{hp})$, and the mean difference in absolute inequality on the y-axis $\:{(GCI}_{p}-\:{GCI}_{i})$, where *i* is treatment group and *p* is the placebo group. We present the trial achievement plane in Fig. 2 below, using the hypothetical distributions in Fig. 1 to explain the plane. In this case the Generalised concentration index is used, however this index can be replaced with another measure of absolute income-related inequality for the purposes of populating the achievement plane (e.g., the Erreygers adjusted index).

Centring the placebo/control group at the origin in Fig. 2, the achievement plane allows the average health effect and distributional impact of each treatment to be assessed simultaneously. Treatments to the right of the origin indicate improved average health, while treatments above the origin indicate reduced pro-rich socioeconomic inequality. Treatments in Quadrant I achieve both — improved average health and reduced inequality — and dominate the control group across the distribution (a “win-win” in the terminology of Cookson et al. [31]), as illustrated by Treatment 2 in Figs. 1 and 2. Treatments in Quadrant III are unfavourable on both dimensions, delivering worse average health and greater inequality, and are dominated by the control group (a “lose-lose” [31]). Treatments in Quadrants II and IV involve trade-offs, favouring either reduced inequality at the expense of average health (Quadrant II) or improved average health at the expense of greater inequality (Quadrant IV). Whether such treatments are preferred to the control depends on the underlying social welfare function; specifically, the weight placed on equity relative to average health gain [17, 30, 31].

### Power calculations for post-hoc inequality testing

Testing for differences in inequality without first ensuring the statistical power of the employed tests risks the possibility of Type II errors. This could be particularly harmful in an inequality context, where underpowered testing may fail to find significant differences in inequality estimates. In post-hoc analyses (including in those reported in this study), an assessment of statistical power is necessary to avoid erroneous conclusions.

As sample sizes are already known and cannot be changed in the post-hoc case, a sensible approach is to test the Minimum Detectable Effect (MDE) and assess how the MDE relates to the investigation context. For example, does the data allow a relevant and appropriate MDE, or does the MDE indicate a lack of precision required for testing the question at hand.

In the context of the concentration index, the $\:{MDE}_{ip}$ which estimates the effect between groups *i* and *p* is given below [32]:

3 $$\:{MDE}_{i-p}=\left({z}_{1-\frac{\alpha\:}{2}}+{z}_{1-\beta\:}\right)*\:SE$$

Where $\:{z}_{1-\frac{\alpha\:}{2}}$ is the critical value for a selected significance value $\:\alpha\:$ for a two-tailed test; $\:{z}_{1-\beta\:}$ is the critical value for selected power $\:\beta\:$, and *SE*$\:\:=SE({I}_{i}-{I}_{p}$) which is the standard error of the calculated concentration indices (*I*) of groups *p* and *i*, where $\:SE\left({I}_{i}-{I}_{p}\right)=\:\sqrt{{{SE}_{Ii}}^{2}+\:{{SE}_{Ip}}^{2}}$ under the assumption of independent groups.

After selecting $\:\alpha\:$ and $\:\beta\:$ values and identifying the appropriate critical values, the MDE between groups *i* and *p* can be easily computed using the standard errors gained from estimating the concentration index (or its extensions). Once the MDEs are estimated, the researcher needs to decide if this is a viable effect size, or if smaller, undetectable effects are crucial. As the test will not pick up smaller effects than the MDE, testing should not continue if smaller differences are critical. An illustration of the process is contained in the empirical application and an MDE reference table with differing combinations of standard errors (SE), significance levels (α), and selected power (β) is included in the supplementary material (Table A1).

## Empirical application & results

### Data description and survey design

To illustrate our approach and show proof-of-concept, we use publicly available data from the Ghana Financial Incentives Trial which investigates the influence of cash incentives on COVID-19 vaccination uptake in six rural Ghanaian districts from 5 February 2022 [8]. The study conducted a two-stage cluster RCT with four treatment arms consisting of a video message embedded in the survey: placebo, CDC-styled informative health message, low cash incentive (20 Ghana cedis / $3) and high cash incentive (60 Ghana cedis / $10). Within each of the six districts, villages were ranked by population and divided into quadruplets consisting of 4 consecutively population ranked villages. Each district had approximately 50 quadruplets, from which 13 were randomly selected, resulting in the inclusion of 310 villages in the study. Within each selected quadruplet, villages were randomly assigned to one of the 4 treatment arms. 21 households were randomly selected within each village, and a single eligible individual over 18 years old was randomly selected from each selected household to complete the survey. Eligibility required non-vaccination at the onset of the trial. 5,900 individuals were treated up to 28th February 2022 [8]. As a measure of socioeconomic status, we use equivalised weekly food expenditure in this study. Respondents were asked, “How much on average does your household spend in a typical week on food?” Food expenditure is equivalised using the OECD modified equivalisation scale, which accounts for the number of adults and children in the household [33]. While a total expenditure variable is also available in the data, this was significantly noisier and food expenditure was preferred. The headline analysis using total expenditure is contained in supplementary Figure A2 and Table A2 as a sensitivity analysis.

Phase 2 and 3 of the trial, conducted from 13 April 2022 and 15 June 2022 respectively, attempted to follow up with Phase 1 participants to collect self-reported vaccination status. 2,082 and 2,019 participants were contacted in phases 2 and 3. Phase 3 also included identification and data collection from 1,101 non-treated, non-placebo allocated individuals in treatment villages for a spillover analysis, which is not included in this paper. Between 15 October and 30 November 2022, staff at district hospitals verified the vaccination status of all respondents to that point. 3,075 total records were verified, including the 2,271 individuals allocated to one of the four primary treatments arms that form the population for this study (placebo, CDC-styled messaging, low-cash incentive, high-cash incentive). The data used in this empirical study is limited to those individuals for whom verified vaccination records were found and who were participants in the treatment phase of the trial, limiting the sample to 2,271 individuals. The full sample characteristics (Table A3), including analysis on self-reported vaccination status, is available in the supplementary materials, and can be compared to the original study.

### Socioeconomic inequality of health outcome indicator

We compute the socioeconomic inequality estimate by treatment arm using the Erreygers adjusted concentration index, a linear transformation of the Generalised concentration index. However, any bounded absolute version of a bivariate inequality index would suffice in this context. The selected measure should fit the data and desired purpose, which may lead to the use of alternative indices [28].

To determine the impact on socioeconomic inequality in vaccine uptake, we compare socioeconomic inequality in each treatment arm against the placebo group [16], as is common in randomised trials. We use 1,000 iterations of bootstrapping resampling in each treatment arm to develop standard errors on the resulting differences. It should be noted that in this proof-of-concept example our bootstrapping procedure resamples at the individual level rather than the village (cluster) level. Given the two-stage cluster design of the original trial, cluster-robust bootstrapping would more fully account for the intra-cluster correlation structure of the data and would be preferable in future applications of this approach to cluster-randomised trials. We visualise these results on the evaluation trials achievement plane to examine the possible trade-offs between socioeconomic equality and uptake. In the concentration index literature, interpretation is often limited to positive, negative, and relative magnitudes. The Erreygers adjusted index is normalised to lie between [-1:1]. Greater absolute index values indicate greater (pro-rich or pro-poor) inequality. In this example *negative (positive)* treatment magnitudes or changes indicate an increase in *pro-rich (pro-poor)* inequality, and values can be compared relative to other treatment magnitudes (e.g., CDC versus high cash incentive). The maximum possible observed difference would thus be 2.0 in the extreme case where the placebo took on a value of -1 and a treatment took on a value of 1, or vice-versa, however in practice such a result is unlikely. In addition, for a comparison of the achievement plane to the standard trial approach we regressed the vaccination status on the treatment arm and equivalised weekly food expenditure with the inclusion of an interaction term. Note that, as the original design was not specifically powered to detect distributional effects, these results and their interpretation should be viewed with caution. There is a risk of both Type I errors from post-hoc analysis, and Type II errors from testing without pre-specified power for the purpose. See sections “Background” and “Power calculations for post-hoc inequality testing” on power calculations and their application to this study.

### Results

#### Balance

In this analysis, we use unadjusted and unweighted verified vaccine status for simplicity and nearest comparison to the original work, however we conduct a check on verified vaccination status using the balanced covariate weightings. Our sample is restricted to respondents with verified vaccination status and complete expenditure (SES) data. In the main work, it is shown that the unadjusted covariates fell within a standardised mean difference tolerance of 0.1 in most instances. We find similarly relative to the placebo group in most cases. Some differences are observed in employment status across treatment groups in our sample. Balances outside the tolerance were also observed in gender and number of children in the household (high cash). The balances are consistent with the main work for the high-cash treatment group; however, the employment imbalances were not previously observed. The weighted differences were balanced, with all but one covariate within the tolerance bounds. The balance details are available in the supplementary materials (Figure A1).

#### Statistical power testing

The process for determining the MDE is outlined in section “Power calculations for post-hoc inequality testing” above, following the formula below, which substitutes $\:SE\left({I}_{i}-{I}_{p}\right)=\:\sqrt{{{SE}_{Ii}}^{2}+\:{{SE}_{Ip}}^{2}}$ from formula 3:

4 $$\:{MDE}_{i-p}=\left({z}_{1-\frac{\alpha\:}{2}}+{z}_{1-\beta\:}\right)*\:\sqrt{{{SE}_{Ii}}^{2}+\:{{SE}_{Ip}}^{2}}$$

In selecting $\:\alpha\:$ and $\:\beta\:$, we select the widely accepted $\:\alpha\:=0.05$ and $\:\beta\:=80\%$, giving critical values of $\:{z}_{1-\frac{\alpha\:}{2}}=1.96$; and $\:{z}_{1-\beta\:}=0.84$, however these values can be altered with rationale relevant to the context (e.g., higher $\:\beta\:$ or lower $\:\alpha\:$ may be desired). All that is now required is to substitute the standard errors of the calculated indices of the respective treatment arms into formula 4. Substituting the values from Table 1 into formula 4, the MDEs are estimated in Table 2.

**Table 1:** Erreygers adjusted concentration index estimates

	Erreygers index	Standard Error
Placebo	-0.098	0.03922
Treatments		
CDC	0.033	0.04470
Low cash	0.013	0.05015
High cash	-0.019	0.04362

**Table 2:** Minimum detectable effects between placebo groups and treatment arms

	$\:\left({z}_{1-\frac{\alpha\:}{2}}+{z}_{1-\beta\:}\right)$	*	$\:\sqrt{{{SE}_{Ii}}^{2}+\:{{SE}_{Ip}}^{2}}$	=	MDE
$\:{MDE}_{CDC-\:p}$	1.96 + 0.84 = 2.8	*	0.05947	=	0.1665
$\:{MDE}_{Low\:cash-p}$	1.96 + 0.84 = 2.8	*	0.06366	**=**	**0.1783**
$\:{MDE}_{High\:cash-p}$	1.96 + 0.84 = 2.8	*	0.05870	**=**	**0.1642**

The calculated MDEs are high in relation to the traditional concentration index literature, where one might want to identify smaller differences between groups. The MDEs indicate that testing will likely only detect true differences between treatment groups and the placebo group with statistical significance if the differences are > 0.17. This shows that in the example of the Ghana Financial Incentives Trial, the trial only has statistical power to detect large distributional shifts. As smaller differences are of interest, the high MDEs calculated here indicate an under-powered test. Without testing for MDEs, null results might be accepted when in fact the test is simply not powered to reject the null hypothesis except when large differences arise. While the test retains value for detecting large differences, if statistical significance is not observed, it can only be used to say that the null hypothesis is not rejected (i.e., differences of < 0.17 between treatments and placebo groups may exist but were not detected). As such, while differences greater than 0.17 can be ruled out with statistical significance, smaller differences cannot be rejected with the data at hand. A sensitivity analysis where $\:\alpha\:$ = 0.1 opposed to 0.05 is included in the supplementary materials as Table A4, where the MDEs decrease, albeit only slightly.

#### Vaccination uptake

Table 3 presents the verified vaccination status per treatment group. At commencement, all individuals are unvaccinated per eligibility requirements. Six weeks after vaccine availability, uptake was relatively similar in the placebo, CDC health message and high-cash groups (within a range of 5 percentage points), but substantially higher in the low-cash group. These figures are within 1–3 percentage points of the original trial work, despite small sample size differences. A reweighting process was conducted on the basis of the balance results above. Using the reweighted figures which balance covariates across treatments, the verified vaccinations proportions were marginally further away from the original trial work than the unweighted estimates, with an average difference of 1.75 percentage points versus 1.5 percentage points for the unweighted estimates. As such, we elect to use the unweighted estimates in the main body of this work, which offers the best approximation of the primary vaccination variable.

**Table 3:** Vaccine sentiment and status at stages of trial

Vaccine sentiment	Placebo	CDC health message	Low cash	High cash
**Commencement**				
Unvaccinated	702 (100%)	488 (100%)	522 (100%)	559 (100%)
**After 6 weeks of availability**				
Verified: Vaccinated	200 (28.4%)	117 (23.9%)	225 (43.1%)	153 (27.3%)
Verified: Not vaccinated	502 (71.6%)	371 (76.1%)	297 (56.9%)	406 (72.7%)
**Duch et al.**,** 2023**				
Verified: Vaccinated	28.4%	21.6%	40.7%	25.9%
**Reweighted for balanced covariates**				
Verified: Vaccinated	28.2%	25.0%	42.4%	28.0%

#### Treatment socioeconomic inequality and uptake trade-offs

**Fig. 3:**
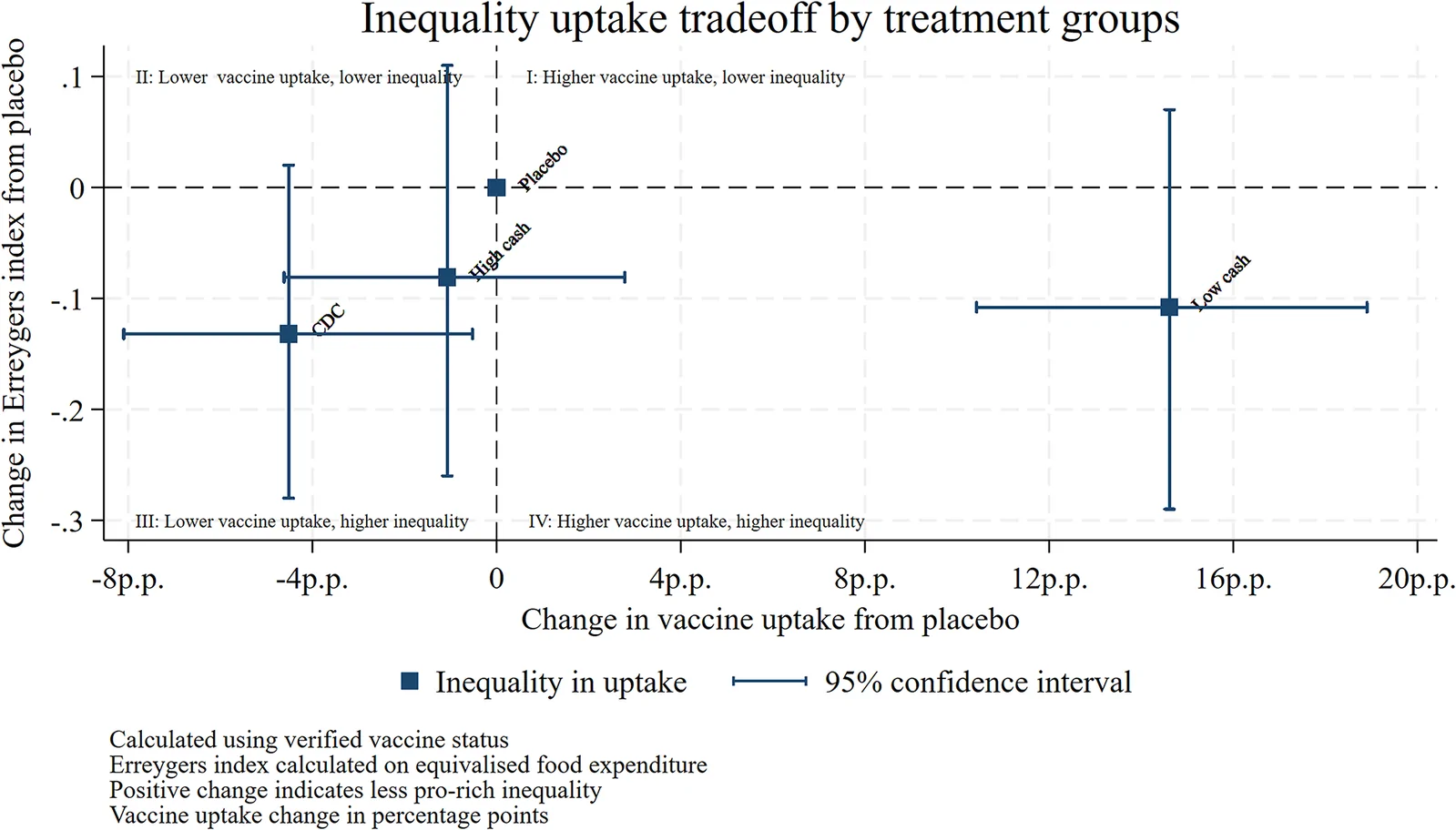
Trials achievement by treatment group using the Erreygers adjusted index

**Fig. 4:**
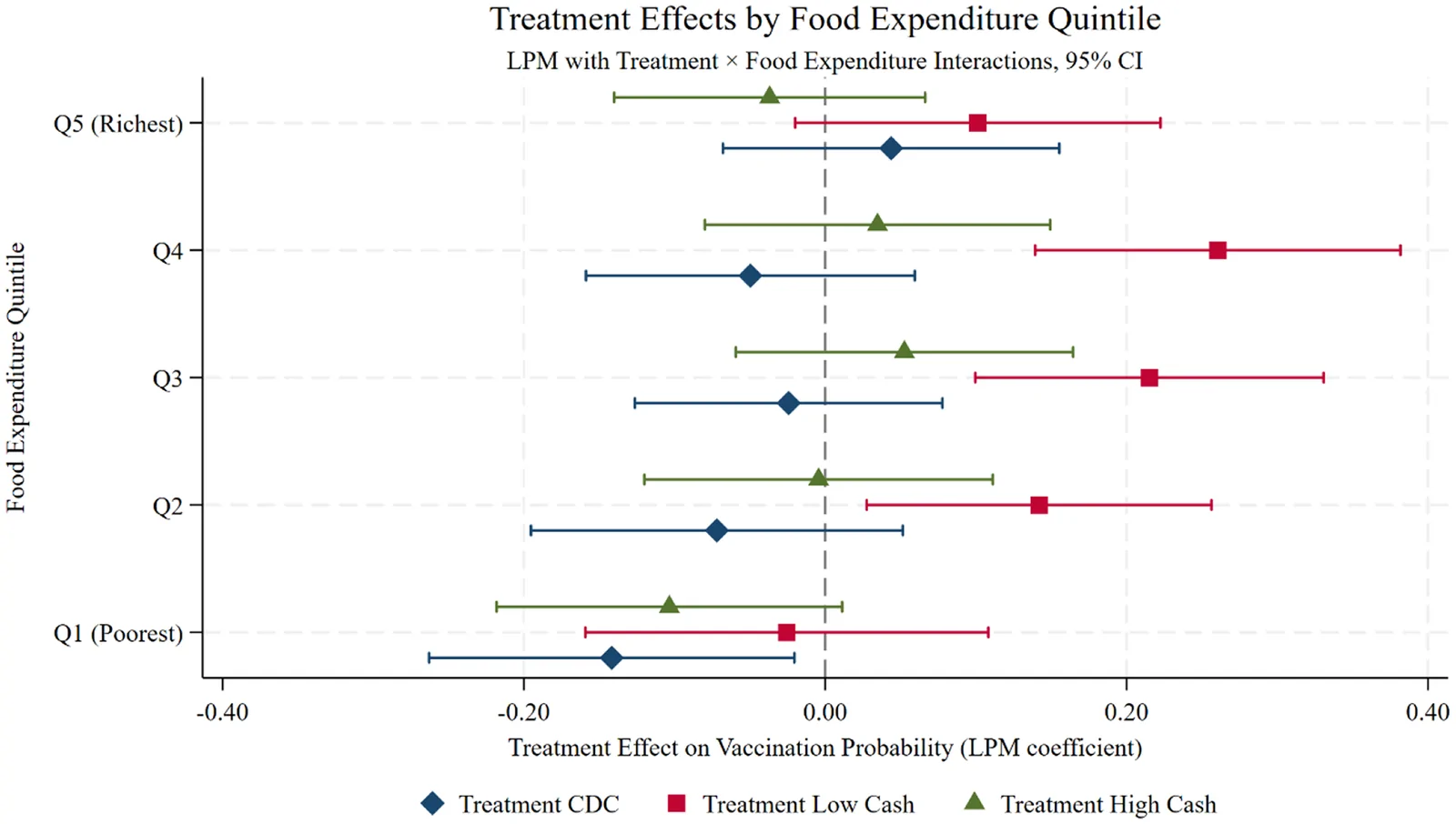
Treatment effects by food expenditure quintile for each treatment arm with a linear probability model

The computed socioeconomic inequality of health indices are presented in Fig. 3; Table 4. The Fig. 3 trials achievement plane visualises the socioeconomic equality uptake trade-off. While no significant differences in socioeconomic inequality of health were observed as shown by treatment effect magnitudes in Table 4, the low-cash treatment had a statistically significantly higher uptake level. The treatment effect magnitudes in Table 4 indicate that there was a non-statistically significant increase in point estimates of pro-rich socioeconomic inequality of health under each treatment, the largest of which (-0.132) was observed in the CDC styled treatment, though remains below the MDE when $$\:\alpha\:=0.05$$ and when $$\:\alpha\:=0.1$$. When vaccination status was regressed on treatment arm and full expenditure no significant differences were found across the expenditure quintiles (Fig. 4). Similarly to the socioeconomic inequality of health indices results, low-cash treatment was observed to have the largest impact on vaccination uptake compared to the placebo. The impact of each treatment arm on vaccine uptake by expenditure quintiles is further visualised in Figure A3. Unlike the socioeconomic inequality of health indices, both the regression analysis and vaccine uptake plot do not capture socioeconomic inequality across the whole distribution but rather are limited to individual quintile groups. While there is not a defined cut-off value at which point policy action is warranted, relative analysis can be conducted that is nuanced and context-appropriate. When all treatments were combined, no significant differences in socioeconomic inequality of health were observed relative to the placebo group. Non-significant differences should not be equated with equivalence, particularly in this study which has large MDEs. In this study, if differences in inequality of less than ~ 0.17 between a given treatment group and the placebo are acceptable to policy makers, then higher uptake can be tentatively prioritised which is achieved by the low-cash treatment. However, smaller distributional differences may exist and the test employed simply does not have the power to detect these differences. In the original work, Duch et al. suggest that the low-cash incentive outperforms the high-cash incentive due to the possibility that the high-cash 60 cedis / $10 incentive (which represents approximately ~ 50% of average weekly food expenditure) may signal a low-quality vaccine and higher potential health risks of the vaccine, to which individuals are averse [8].

**Table 4:** Bootstrapped treatment effects using verified vaccine status

	Erreygers index	Treatment effect magnitude^1^ [95% CI]^2^
Placebo	-0.099	
Treatments		
CDC	0.033	-0.132 [-0.28–0.02]
Low cash	0.009	-0.108 [-0.29–0.07]
High cash	-0.019	-0.081 [-0.26–0.11]

Notes

1. Treatment magnitude refers to the average difference between inequality of treated in treatment villages and inequality of untreated in untreated villages 6 weeks after vaccine availability. i.e., placebo index – treatment index

2. *** *p* < 0.001, ** *p* < 0.01, * *p *< 0.05

3. Indices vary slightly compared to Table 1 due to bootstrapping sampling differences

## Discussion

In this paper we have demonstrated how socioeconomic inequality indices can be integrated into the reporting of randomised experiments alongside traditional outcome measures such as average treatment effects. Using an illustrative empirical application drawn from a COVID-19 vaccination uptake trial in Ghana during the pandemic, we have shown how the achievement plane can be used to jointly characterise the efficiency and distributional impacts of competing interventions, providing a richer basis for decision-making than average treatment effects alone. The results from the Ghana trial indicated no evidence of significant differences in the socioeconomic distributional impacts between the placebo and treatment groups. On the basis of the available evidence, treatments with the greatest average uptake, in this case the low-cash incentive, would therefore be prioritised, as there is limited evidence of large distributional differences between treatments.

Policy makers routinely face decisions that require balancing efficiency against equity considerations, of which socioeconomic inequality in health is one important dimension. The achievement plane provides a natural framework for structuring this trade-off, placing both dimensions on a common footing and making the nature of any equity-efficiency tension explicit. As with any analytical tool applied post-hoc to trial data, conclusions should be drawn with appropriate care, and the substantive significance of observed differences considered alongside their statistical properties. Nonetheless, even where firm conclusions cannot be drawn, the framework usefully clarifies what the data can and cannot tell us about the distributional implications of an intervention.

A natural question is how the approach proposed here relates to the heterogeneity analyses that are routinely reported in RCTs. Subgroup and interaction analyses are increasingly common in trial reporting, and socioeconomic status is sometimes included as one of several effect modifiers examined (see Fig. 4 as an illustrative example). However, traditional heterogeneity testing has important limitations as a tool for equity analysis. Subgroup analyses typically test whether the average treatment effect differs across broad, discretely defined groups; for example, comparing effects in the poorest and wealthiest quintiles and in doing so discard much of the information contained in the continuous socioeconomic distribution. In contrast, the concentration index uses the full distribution of socioeconomic status across all trial participants, weighting health outcomes by relative income rank and thereby capturing the gradient of inequality across the entire spectrum rather than at a small number of arbitrarily defined cut-off points. This makes it a more sensitive and information-efficient summary of the socioeconomic distribution of health. The achievement plane builds on this by jointly presenting the average treatment effect and the distributional impact within a common framework, making the equity-efficiency trade-off explicit and comparable across interventions. Rather than treating equity as one among many secondary subgroup analyses, the achievement plane elevates it to a co-equal dimension of trial evaluation alongside average effectiveness.

The Ghana vaccination trial represents an important proof-of-concept for the methods proposed in this paper. Conducted during the COVID-19 pandemic, the trial addresses a highly policy-relevant question: whether financial incentives can increase vaccine uptake, and what the distributional consequences of such incentives are across the socioeconomic spectrum. Equitable vaccine distribution was widely recognised as a critical challenge during the pandemic, with concerns that uptake incentives could differentially benefit wealthier or better-connected populations, inadvertently widening rather than narrowing health inequalities. The collection of socioeconomic data alongside health outcomes is precisely the kind of trial design that enables the achievement plane to be applied, and the multi-arm structure – comparing cash incentives of different magnitudes against a placebo – allows the equity-efficiency trade-off to be illustrated across several interventions simultaneously. The achievement plane makes this potential tension visible in a way that reporting average treatment effects alone does not.

At the same time, a number of limitations should be noted when interpreting the inequality statistics reported here. The original trial authors identified several constraints on generalisability: potential discrepancies in the village-level rollout of vaccination; results that may be specific to the study design; the exclusively rural sample; and unknown spillover effects under scaling. These cautions apply with particular force to the socioeconomic inequality estimates. The concentration index captures the distribution of health across the socioeconomic spectrum within the study sample, and in a rural-only sample this distribution may differ substantially from that of the broader Ghanaian population or other settings. The trial’s inequality statistics should therefore not be generalised beyond the study context, and their application to wider policy should be approached with care.

Four avenues for future work are worth noting. First, applying explicit social welfare foundations to the achievement plane would enrich its analytical basis. As Bleichrodt and van Doorslaer (2006) have shown, the achievement index is the abbreviated social welfare function underlying the extended concentration index, and the plane currently assumes equal weighting on average health and inequality [34]. Further work linking social welfare properties to the achievement plane would enable both the selection of interventions within the same quadrant and, where appropriate, the redrawing of quadrant boundaries according to the underlying social welfare function, since outcomes in Quadrants II or IV may be preferred to those in Quadrant I depending on normative assumptions. Simulated RCT data with a variety of known distributional effects would be a valuable tool for this work, allowing each hypothetical scenario and underlying social welfare function to be illustrated directly, and helping to inform sample size calculations for future trials that aim to prospectively power distributional testing. Second, the application of joint distributions of health outcomes and socioeconomic inequality could support the development of an acceptability curve analogous to that used in cost-effectiveness analysis, enabling decision-making that incorporates both dimensions simultaneously. Fleurbaey’s work combining health and income into a single distribution using a willingness-to-pay metric offers a promising foundation for this extension [35]. Third, and particularly promising, is the integration of this framework with the growing literature on distributional cost-effectiveness analysis (DCEA). DCEA extends conventional economic evaluation by weighting health outcomes according to the socioeconomic position of recipients, and there is a natural complementarity between the achievement plane and DCEA methods, both of which are concerned with making the distributional consequences of interventions explicit and tractable for decision-makers. In fact, the achievement index (formula 2) is a potential way to adjust outcomes to account for distribution effects in DCEA. Linking trial-based inequality measurement to DCEA frameworks would provide a more complete pipeline from trial evidence to equity-informed resource allocation decisions. A fourth avenue concerns the extension of this framework beyond randomised trials to quasi-experimental designs. While the RCT setting provides a particularly clean basis for causal inference about distributional effects, the underlying logic extends naturally to other evaluation designs. In difference-in-differences analyses, for example, the parallel trends assumption plays an analogous role to randomisation, and concentration indices estimated on treated and control groups before and after the intervention could similarly be used to recover a causal distributional treatment effect. Extending the framework to these and other quasi-experimental settings such as regression discontinuity and instrumental variable designs would substantially broaden its applicability, since many important policy evaluations rely on quasi-experimental rather than experimental variation.

In summary, this paper has demonstrated how the achievement plane can be used to jointly characterise the socioeconomic equality and efficiency implications of competing interventions in randomised trials. Treatment effects on the socioeconomic distribution of health are an important complement to average treatment effects, and their routine reporting would better equip policy makers to avoid equality-blind decisions that risk inadvertently widening health inequalities. The Ghana trial illustrates both the value of collecting socioeconomic data alongside trial outcomes and the analytical insights that become available when this is done, insights that average treatment effects alone cannot provide.

## Supplementary Information

Below is the link to the electronic supplementary material.

Supplementary Material 1

## Data Availability

All anonymized data used in the analysis are publicly and freely available at https://doi.org/10.7910/DVN/X5MBHP.

## Declarations

### Use of AI assistance

During the preparation of this manuscript, the authors used Claude (Anthropic) to assist with drafting and editing text. All AI-generated or AI-assisted content was reviewed, revised, and approved by the authors, who take full responsibility for the accuracy and integrity of the published work. AI assistance was not used in the design or conduct of the analysis, the interpretation of results, or the development of the methodological framework.

## References

[1] Commonwealth Fund, Mirror M. 2021: Reflecting Poorly [Internet]. 2021 Aug [cited 2023 Nov 17]. Available from: https://www.commonwealthfund.org/publications/fund-reports/2021/aug/mirror-mirror-2021-reflecting-poorly

[2] Avanceña ALV, Prosser LA. Examining Equity Effects of Health Interventions in Cost-Effectiveness Analysis: A Systematic Review. Value Health. 2021;24(1):136–43. 10.1016/j.jval.2020.10.010. 33431148

[3] Patikorn C, Cho JY, Lambach P, Hutubessy R, Chaiyakunapruk N. Equity-Informative Economic Evaluations of Vaccines: A Systematic Literature Review. Vaccines. 2023;11(3):3. 10.3390/vaccines11030622. PMC10057152 36992206

[4] Ward T, Mujica-Mota RE, Spencer AE, Medina-Lara A. Incorporating Equity Concerns in Cost-Effectiveness Analyses: A Systematic Literature Review. PharmacoEconomics. 2022;40(1):45–64. 10.1007/s40273-021-01094-7. 34713423

[5] Endawkie A, Daba C, Asmare L, Desye B, Mawugatie TW, Melak D, et al. Trends of socioeconomic and geographic inequalities in COVID-19 vaccine uptake in Ethiopia: using the WHO Health Equity Assessment Toolkit. BMC Health Serv Res. 2024;24(1):1629. 10.1186/s12913-024-12082-w. PMC11660808 39707307

[6] Abel ZD, Roope LS, Duch R, Cole S, Clarke PM. Inequality in COVID-19 vaccine acceptance and uptake: A repeated cross-sectional analysis of COVID vaccine acceptance and uptake in 13 countries. Health Policy. 2025;153:105251. 10.1016/j.healthpol.2025.105251. PubMed PMID: 39862594. 39862594

[7] Niño L, Kiragga A, Miller FD, Mwalili SM, Musuka G, Cuadros DF. Mapping structural barriers: a geospatial assessment of COVID-19 vaccine inequities in Kenya. medRxiv [Preprint]. 2025 Apr 14:2025.04.14.25325797.

[8] Duch R, Asiedu E, Nakamura R, Rouyard T, Mayol A, Barnett A, et al. Financial incentives for COVID-19 vaccines in a rural low-resource setting: a cluster-randomized trial. Nat Med. 2023;29(12):12. 10.1038/s41591-023-02670-4. PMC10719106 38012315

[9] UN General Assembly. Transforming our world: the 2030 Agenda for Sustainable Development [General Assembly Resolution A/RES/70/1.] [Internet]. New York: United Nations. 2015 Oct [cited 2024 May 10]. Available from: https://www.refworld.org/legal/resolution/unga/2015/en/111816

[10] Deaton A, Cartwright N. Understanding and misunderstanding randomized controlled trials [Internet]. Cambridge (MA): National Bureau of Economic Research; 2016 Sep [cited 2026 Jun 8]. (NBER Working Paper; No. 22595). Available from: http://www.nber.org/papers/w22595.pdf. .

[11] Petkovic J, Jull J, Yoganathan M, Dewidar O, Baird S, Grimshaw JM, et al. Reporting of health equity considerations in cluster and individually randomized trials. Trials. 2020;21(1):308. 10.1186/s13063-020-4223-5. PMC7118943 32245522

[12] Ong SWX, Blagojevic C, Bryce A, Ovadia A, Slater M, Pryal D, et al. Reporting of sociodemographic characteristics of trial participants in infectious diseases clinical trials-a systematic review. Clin Microbiol Infect. 2025;31(8):1283–90. 10.1016/j.cmi.2025.04.030 . PubMed PMID: 40339794. 40339794

[13] Welch VA, Norheim OF, Jull J, Cookson R, Sommerfelt H, Tugwell P, et al. CONSORT-Equity 2017 extension and elaboration for better reporting of health equity in randomised trials. BMJ. 2017;359:j5085. 10.1136/bmj.j5085 . PubMed PMID: 29170161. 29170161

[14] Cookson R, Griffin S, Norheim OF, Culyer AJ, Chalkidou K. Distributional Cost-Effectiveness Analysis Comes of Age. Value Health. 2021;24(1):118–20. 10.1016/j.jval.2020.10.001 . PubMed PMID: 33431145; PubMed Central PMCID: PMC7813213. PMC7813213 33431145

[15] Ghasempour M, de Luna X, Gustafsson PE. Causal inference targeting a concentration index for studies of health inequalities [Internet]. arXiv; 2025 [cited 2026 Jun 8]. Available from: http://arxiv.org/abs/2410.08849. 10.1093/biomtc/ujag082 42145179

[16] Choudhary TS, Mazumder S, Haaland ØA, Taneja S, Bahl R, Martines J, et al. Health equity impact of community-initiated kangaroo mother care: a randomized controlled trial. Int J Equity Health. 2021;20(1):263. 10.1186/s12939-021-01605-0. PMC8709992 34952592

[17] Clarke PM, Gerdtham UG, Johannesson M, Bingefors K, Smith L. On the measurement of relative and absolute income-related health inequality. Soc Sci Med. 2002;55(11):1923–8. 10.1016/S0277-9536(01)00321-5. 12406461

[18] Erreygers G, Clarke P, Van Ourti T. Mirror, mirror, on the wall, who in this land is fairest of all?—Distributional sensitivity in the measurement of socioeconomic inequality of health. J Health Econ. 2012;31(1):257–70. 10.1016/j.jhealeco.2011.10.009. PMC3349439 22204878

[19] Harper S, King NB, Meersman SC, Reichman ME, Breen N, Lynch J. Implicit Value Judgments in the Measurement of Health Inequalities. Milbank Q. 2010;88(1):4–29. 10.1111/j.1468-0009.2010.00587. .x PubMed PMID: 20377756; PubMed Central PMCID: PMC2888011. PMC2888011 20377756

[20] Schlotheuber A, Hosseinpoor AR. Summary Measures of Health Inequality: A Review of Existing Measures and Their Application. Int J Environ Res Public Health. 2022;19(6):3697. 10.3390/ijerph19063697 . PubMed PMID: 35329383; PubMed Central PMCID: PMC8992138. PMC8992138 35329383

[21] Chen X, Nordhaus WD. Using luminosity data as a proxy for economic statistics. Proc Natl Acad Sci USA. 2011;108(21):8589–94. 10.1073/pnas.1017031108 . PubMed PMID: 21576474; PubMed Central PMCID: PMC3102367. PMC3102367 21576474

[22] Jerrim J. Measuring socio-economic background using administrative data. What is the best proxy available? DoQSS Working Papers [Internet]. 2020 Aug 1 [cited 2024 Jun 11];DoQSS Working Papers:20–09. Available from: https://ideas.repec.org//p/qss/dqsswp/2009.html

[23] Kakwani NC. Income inequality and poverty: methods of estimation and policy applications [Internet]. Published for the World Bank by Oxford University Press; 1980 [cited 2023 Nov 22]. Available from: https://digitallibrary.un.org/record/50

[24] Wagstaff A, Paci P, van Doorslaer E. On the measurement of inequalities in health. Soc Sci Med. 1991;33(5):545–57. 10.1016/0277-9536. (91)90212-u PubMed PMID: 1962226. 1962226

[25] Wagstaff A. The bounds of the concentration index when the variable of interest is binary, with an application to immunization inequality. Health Econ. 2005;14(4):429–32. 10.1002/hec.953. 15495147

[26] Erreygers G. Correcting the concentration index. J Health Econ. 2009;28(2):504–15. 10.1016/j.jhealeco.2008. 02.003 PubMed PMID: 18367273. 18367273

[27] Erreygers G, Kessels R. Socioeconomic Status and Health: A New Approach to the Measurement of Bivariate Inequality. Int J Environ Res Public Health. 2017;14(7):673. 10.3390/ijerph14070673 . PubMed PMID: 28644405; PubMed Central PMCID: PMC5551111. PMC5551111 28644405

[28] Erreygers G, Van Ourti T. Measuring socioeconomic inequality in health, health care and health financing by means of rank-dependent indices: A recipe for good practice. J Health Econ. 2011;30(4):685–94. 10.1016/j.jhealeco.2011.04.004. PMC3158909 21683462

[29] Wagstaff A. Inequality aversion, health inequalities and health achievement. J Health Econ. 2002;21(4):627–41. 10.1016/S0167-6296(02)00006-1. 12146594

[30] Clarke PM, Hayes AJ. Measuring achievement: Changes in risk factors for cardiovascular disease in Australia. Soc Sci Med. 2009;68(3):552–61. 10.1016/j.socscimed.2008.09.065. 19038484

[31] Cookson R, Mirelman AJ, Griffin S, Asaria M, Dawkins B, Norheim OF, et al. Using Cost-Effectiveness Analysis to Address Health Equity Concerns. Value Health. 2017;20(2):206–12. 10.1016/j.jval.2016.11.027. PMC5340318 28237196

[32] Djimeu EW, Houndolo DG. Power calculation for causal inference in social science: sample size and minimum detectable effect determination. J Dev Eff. 2016;8(4):508–27. 10.1080/19439342.2016.1244555.

[33] OECD. OECD Framework for Statistics on the Distribution of Household Income. Consumption and Wealth [Internet]. Paris: Organisation for Economic Co-operation and Development; 2013 [cited 2024 Jun 11]. Available from: https://www.oecd-ilibrary.org/economics/framework-for-statistics-on-the-distribution-of-household-income-consumption-and-wealth_9789264194830-en

[34] Bleichrodt H, van Doorslaer E. A welfare economics foundation for health inequality measurement. J Health Econ. 2006;25(5):945–57. 10.1016/j.jhealeco.2006.01.002. 16466818

[35] Fleurbaey M. Health, Wealth, and Fairness. J Public Econ Theor. 2005;7(2):253–84. 10.1111/j.1467-9779.2005.00203.x.

