# Supplementary material for: Moving beyond the average: a method to measure health related-inequalities within randomised trials
Source: Int J Equity Health. 2026 Sep 29;25:225. doi: 10.1186/s12939-026-02977-x (PMC13621616; doi:10.1186/s12939-026-02977-x)
Supplement: Supplementary file 1 [file 12939_2026_2977_MOESM1_ESM.docx]

**Supplementary materials**

Figure A1: Covariate balance table

 Figure A2: Estimated inequality uptake trade-off disaggregated by treatment arm using Erreygers adjusted index and total expenditure

Figure A3: Percentage vaccination uptake across each expenditure quintile and treatment arm

Table A1: Minimum Detectable Effects (MDE) by Standard Error, Significance Level, and Statistical Power (Two-Tailed Test)

| SE (×10−²) | α = 0.10 | | | | α = 0.05 | | | | α = 0.01 | | | |
| --- | --- | --- | --- | --- | --- | --- | --- | --- | --- | --- | --- | --- |
|  | z₁₋α/₂ = 1.645 | | | | z₁₋α/₂ = 1.960 | | | | z₁₋α/₂ = 2.576 | | | |
|  | 1−β = 0.70 | 1−β = 0.80 | 1−β = 0.90 | 1−β = 0.95 | 1−β = 0.70 | 1−β = 0.80 | 1−β = 0.90 | 1−β = 0.95 | 1−β = 0.70 | 1−β = 0.80 | 1−β = 0.90 | 1−β = 0.95 |
| 0.50% | 1.08% | 1.24% | 1.46% | 1.65% | 1.24% | 1.40% | 1.62% | 1.80% | 1.55% | 1.71% | 1.93% | 2.11% |
| 1.00% | 2.17% | 2.49% | 2.93% | 3.29% | 2.48% | 2.80% | 3.24% | 3.60% | 3.10% | 3.42% | 3.86% | 4.22% |
| 1.50% | 3.25% | 3.73% | 4.39% | 4.94% | 3.73% | 4.20% | 4.86% | 5.41% | 4.65% | 5.13% | 5.79% | 6.33% |
| 2.00% | 4.34% | 4.97% | 5.85% | 6.58% | 4.97% | 5.60% | 6.48% | 7.21% | 6.20% | 6.84% | 7.72% | 8.44% |
| 2.50% | 5.42% | 6.22% | 7.32% | 8.22% | 6.21% | 7.00% | 8.11% | 9.01% | 7.75% | 8.54% | 9.64% | 10.55% |
| 3.00% | 6.51% | 7.46% | 8.78% | 9.87% | 7.45% | 8.41% | 9.73% | 10.81% | 9.30% | 10.25% | 11.57% | 12.66% |
| 3.50% | 7.59% | 8.70% | 10.24% | 11.52% | 8.69% | 9.81% | 11.35% | 12.62% | 10.85% | 11.96% | 13.50% | 14.77% |
| 4.00% | 8.68% | 9.95% | 11.71% | 13.16% | 9.94% | 11.21% | 12.97% | 14.42% | 12.40% | 13.67% | 15.43% | 16.88% |
| 4.50% | 9.76% | 11.19% | 13.17% | 14.80% | 11.18% | 12.61% | 14.59% | 16.22% | 13.95% | 15.38% | 17.36% | 18.99% |
| 5.00% | 10.85% | 12.44% | 14.63% | 16.45% | 12.42% | 14.01% | 16.21% | 18.02% | 15.50% | 17.09% | 19.29% | 21.11% |
| 5.50% | 11.93% | 13.68% | 16.10% | 18.09% | 13.66% | 15.41% | 17.83% | 19.83% | 17.05% | 18.80% | 21.22% | 23.22% |
| 6.00% | 13.01% | 14.92% | 17.56% | 19.74% | 14.90% | 16.81% | 19.45% | 21.63% | 18.60% | 20.51% | 23.15% | 25.33% |
| 6.50% | 14.10% | 16.17% | 19.03% | 21.39% | 16.15% | 18.21% | 21.07% | 23.43% | 20.15% | 22.22% | 25.08% | 27.44% |
| 7.00% | 15.18% | 17.41% | 20.49% | 23.03% | 17.39% | 19.61% | 22.69% | 25.24% | 21.70% | 23.93% | 27.01% | 29.55% |
| 7.50% | 16.27% | 18.65% | 21.95% | 24.68% | 18.63% | 21.02% | 24.32% | 27.04% | 23.25% | 25.64% | 28.94% | 31.66% |
| 8.00% | 17.35% | 19.90% | 23.42% | 26.32% | 19.87% | 22.42% | 25.94% | 28.84% | 24.80% | 27.34% | 30.86% | 33.77% |
| 8.50% | 18.44% | 21.14% | 24.88% | 27.96% | 21.11% | 23.82% | 27.56% | 30.64% | 26.35% | 29.05% | 32.79% | 35.88% |
| 9.00% | 19.52% | 22.38% | 26.34% | 29.61% | 22.36% | 25.22% | 29.18% | 32.45% | 27.90% | 30.76% | 34.72% | 37.99% |
| 9.50% | 20.61% | 23.63% | 27.81% | 31.25% | 23.60% | 26.62% | 30.80% | 34.25% | 29.45% | 32.47% | 36.65% | 40.10% |
| 10.00% | 21.69% | 24.87% | 29.27% | 32.90% | 24.84% | 28.02% | 32.42% | 36.05% | 31.00% | 34.18% | 38.58% | 42.21% |
|  |  |  |  |  |  |  |  |  |  |  |  |  |

Note.

1. Formula: MDE = (z₁₋α/₂ + z₁₋β) × SE, where z₁₋α/₂ is the two-tailed critical value for significance level α and z₁₋β is the critical value corresponding to power 1−β.
2. SE denotes the standard error of the estimator.
3. All MDE values are expressed in percentage points (pp).
4. Critical values: α = 0.10 → z = 1.645; α = 0.05 → z = 1.960; α = 0.01 → z = 2.576.
5. Power values: 1−β = 0.70 → z = 0.524; 1−β = 0.80 → z = 0.842; 1−β = 0.90 → z = 1.282; 1−β = 0.95 → z = 1.645.

Table A2: Bootstrapped treatment effects using verified vaccine status on Erreygers adjusted concentration index with total expenditure

|  | Erreygers Concentration index | Treatment magnitude^1^ [95% CI]^2^ |  |
| --- | --- | --- | --- |
| Placebo | -0.075 |  |  |
| Treatments |  |  |  |
| CDC | 0.026 | -0.103 [-0.25 - 0.04] |  |
| Low cash | 0.031 | -0.105 [-0.30 - 0.09] |  |
| High cash | -0.002 | -0.072 [-0.25 - 0.11] |  |
| Notes   1. Treatment magnitude refers to the average difference between inequality of treated in treatment villages and inequality of untreated in untreated villages 6 weeks after vaccine availability 2. *** p<0.001, ** p<0.01, * p<0.05 | | | |

Table A3: Sample characteristics of individuals with verified vaccination status

|  |  | Placebo | CDC | Low cash | High cash |
| --- | --- | --- | --- | --- | --- |
|  | Sample size | 702 | 488 | 522 | 559 |
| Age | Age (mean) | 36.2 | 35.1 | 36.1 | 37.5 |
|  | Age (SD) | 15.5 | 15.3 | 15.5 | 16.8 |
| Gender | Male | 327 | 213 | 233 | 217 |
|  | Female | 375 | 275 | 289 | 341 |
|  | Other gender |  |  |  | 1 |
| Household | HH size (mean) | 6.2 | 6 | 6.2 | 6.4 |
|  | HH size (SD) | 2.2 | 3.3 | 2.8 | 2.7 |
|  | Children in HH (mean) | 1.8 | 1.7 | 1.9 | 2. |
|  | Children in HH (SD) | 1.6 | 1.7 | 1.7 | 1.8 |
| Education | No Schooling | 95 | 69 | 85 | 103 |
|  | Pre-school | 5 | 1 | 8 | 5 |
|  | Primary School | 113 | 83 | 103 | 88 |
|  | Junior Secondary | 359 | 220 | 239 | 285 |
|  | Secondary | 104 | 99 | 75 | 65 |
|  | Tertiary education | 26 | 16 | 12 | 13 |
| Employment | Employed full-time | 395 | 274 | 311 | 295 |
|  | Employed part-time | 119 | 51 | 60 | 96 |
|  | Retired | 16 | 19 | 19 | 20 |
|  | Student | 49 | 46 | 26 | 37 |
|  | Unemployed | 100 | 67 | 79 | 68 |
| Living arrangements | Does not live with partner | 262 | 205 | 206 | 197 |
|  | Live with partner | 232 | 157 | 164 | 182 |
| Expenditure | Food spend (mean) | 163.4 | 164.8 | 164.1 | 164.6 |
|  | Food spend (SD) | 99.5 | 98.2 | 96.6 | 103.2 |
|  | Non-food spend (mean) | 35.1 | 81.3 | 41.2 | 36.6 |
|  | Non-food spend (SD) | 71. | 558.4 | 57.8 | 55.8 |
| Vaccination | Unvaccinated at commencement | 702 | 488 | 522 | 559 |
|  | Vaccine intent | 499 | 350 | 430 | 445 |
|  | Vaccine hesitant | 176 | 130 | 81 | 90 |
|  | Not collected / prefer not say | 27 | 8 | 11 | 24 |
|  | Verified: Vaccinated (6 weeks) | 200 | 117 | 225 | 153 |
|  | Verified: Not vaccinated (6 weeks) | 502 | 371 | 297 | 406 |
|  | Reported: Vaccinated | 203 | 155 | 166 | 165 |
|  | Change in vaccine uptake (p.p.) |  | -4.5 | 14.6 | -1.1 |

Table A4: Minimum detectable effects where $\alpha$=0.1

|  | $\left( z_{1-\frac{\alpha}{2}}+z_{1-\beta} \right)$ | * | $\sqrt{{{SE}_{Ii}}^{2}+ {{SE}_{Ip}}^{2}}$ | **=** | **MDE** |
| --- | --- | --- | --- | --- | --- |
| ${MDE}_{CDC- p}$ | 1.645 + 0.84 = 2.485 | * | 0.05947 | **=** | **0.1478** |
|  |  |  |  |  |  |
| ${MDE}_{Low cash-p}$ | 1.645 + 0.84 = 2.485 | * | 0.06366 | **=** | **0.1582** |
|  |  |  |  |  |  |
| ${MDE}_{High cash-p}$ | 1.645 + 0.84 = 2.485 | * | 0.05870 | **=** | **0.1459** |
| Note: where $\alpha$=0.1 and $\beta$=0.8 | | | | | |
